# Immunogenicity of Severe Acute Respiratory Syndrome Coronavirus 2 (SARS-CoV-2) Infection and Ad26.CoV2.S Vaccination in People Living With Human Immunodeficiency Virus (HIV)

**DOI:** 10.1093/cid/ciab1008

**Published:** 2021-12-10

**Authors:** Khadija Khan, Gila Lustig, Mallory Bernstein, Derseree Archary, Sandile Cele, Farina Karim, Muneerah Smith, Yashica Ganga, Zesuliwe Jule, Kajal Reedoy, Yoliswa Miya, Ntombifuthi Mthabela, Nombulelo P Magula, Richard Lessells, Tulio de Oliveira, Bernadett I Gosnell, Salim Abdool Karim, Nigel Garrett, Willem Hanekom, Linda-Gail Bekker, Glenda Gray, Jonathan M Blackburn, Mahomed-Yunus S Moosa, Alex Sigal, COMMIT-KZN Team, Adrie Steyn, Alasdair Leslie, Dirhona Ramjit, Emily Wong, Guy Harling, Henrik Kloverpris, Jackson Marakalala, Janet Seeley, Jennifer Giandhari, Kaylesh Dullabh, Kennedy Nyamande, Kobus Herbst, Kogie Naidoo, Matilda Mazibuko, Moherndran Archary, Mosa Moshabela, Nesri Padayatchi, Nigel Klein, Nikiwe Mbatha, Nokuthula Ngcobo, Nokwanda Gumede, Nokwanda Ngcobo, Philip Goulder, Prakash Jeena, Rajhmun Madansein, Ravindra K. Gupta, Rohen Harrichandparsad, Samita Singh, Thandeka Khoza, Theresa Smit, Thumbi Ndung'u, Vinod Patel, Zaza Ndhlovu

**Affiliations:** Africa Health Research Institute, Durban, South Africa; School of Laboratory Medicine and Medical Sciences, University of KwaZulu-Natal, Durban, South Africa; Centre for the AIDS Programme of Research in South Africa, Durban, South Africa; Africa Health Research Institute, Durban, South Africa; Centre for the AIDS Programme of Research in South Africa, Durban, South Africa; Department of Medical Microbiology, University of KwaZulu-Natal, Durban, South Africa; Africa Health Research Institute, Durban, South Africa; School of Laboratory Medicine and Medical Sciences, University of KwaZulu-Natal, Durban, South Africa; Africa Health Research Institute, Durban, South Africa; School of Laboratory Medicine and Medical Sciences, University of KwaZulu-Natal, Durban, South Africa; Department of Integrative Biomedical Sciences, Faculty of Health Sciences, University of Cape Town, Cape Town, South Africa; Africa Health Research Institute, Durban, South Africa; Africa Health Research Institute, Durban, South Africa; Africa Health Research Institute, Durban, South Africa; Africa Health Research Institute, Durban, South Africa; Africa Health Research Institute, Durban, South Africa; Department of Medicine, King Edward VIII Hospital and University of KwaZulu Natal, Durban, South Africa; School of Laboratory Medicine and Medical Sciences, University of KwaZulu-Natal, Durban, South Africa; Centre for the AIDS Programme of Research in South Africa, Durban, South Africa; KwaZulu-Natal Research Innovation and Sequencing Platform, Durban, South Africa; School of Laboratory Medicine and Medical Sciences, University of KwaZulu-Natal, Durban, South Africa; Centre for the AIDS Programme of Research in South Africa, Durban, South Africa; KwaZulu-Natal Research Innovation and Sequencing Platform, Durban, South Africa; Centre for Epidemic Response and Innovation, School of Data Science and Computational Thinking, Stellenbosch University, Stellenbosch, South Africa; Department of Global Health, University of Washington, Seattle, Washington, USA; Department of Infectious Diseases, Nelson R. Mandela School of Clinical Medicine, University of KwaZulu-Natal, Durban, South Africa; Centre for the AIDS Programme of Research in South Africa, Durban, South Africa; Department of Epidemiology, Mailman School of Public Health, Columbia University, New York, New York, USA; Centre for the AIDS Programme of Research in South Africa, Durban, South Africa; Discipline of Public Health Medicine, School of Nursing and Public Health, University of KwaZulu-Natal, Durban, South Africa; Africa Health Research Institute, Durban, South Africa; Division of Infection and Immunity, University College London, London, United Kingdom; Institute of Infectious Disease and Molecular Medicine, University of Cape Town, Cape Town, South Africa; Desmond Tutu HIV Centre, Cape Town, South Africa; South African Medical Research Council, Cape Town, South Africa; Department of Integrative Biomedical Sciences, Faculty of Health Sciences, University of Cape Town, Cape Town, South Africa; Institute of Infectious Disease and Molecular Medicine, University of Cape Town, Cape Town, South Africa; Sengenics Corporation, Kuala Lumpur, Malaysia; Department of Infectious Diseases, Nelson R. Mandela School of Clinical Medicine, University of KwaZulu-Natal, Durban, South Africa; Africa Health Research Institute, Durban, South Africa; School of Laboratory Medicine and Medical Sciences, University of KwaZulu-Natal, Durban, South Africa

**Keywords:** SARS-CoV-2, Ad26.CoV2.S vaccines, immunogenicity, neutralization, HIV viremia

## Abstract

**Background:**

People living with HIV (PLWH) have been reported to have a higher risk of more severe COVID-19 disease and death. We assessed the ability of the Ad26.CoV2.S vaccine to elicit neutralizing activity against the Delta variant in PLWH relative to HIV-negative individuals. We also examined effects of HIV status and suppression on Delta neutralization response in SARS-CoV-2—infected unvaccinated participants.

**Methods:**

We enrolled participants who were vaccinated through the SISONKE South African clinical trial of the Ad26.CoV2.S vaccine in healthcare workers (HCWs). PLWH in this group had well-controlled HIV infection. We also enrolled unvaccinated participants previously infected with SARS-CoV-2. Neutralization capacity was assessed by a live virus neutralization assay of the Delta variant.

**Results:**

Most Ad26.CoV2.S vaccinated HCWs were previously infected with SARS-CoV-2. In this group, Delta variant neutralization was 9-fold higher compared with the infected-only group and 26-fold higher relative to the vaccinated-only group. No decrease in Delta variant neutralization was observed in PLWH relative to HIV-negative participants. In contrast, SARS-CoV-2—infected, unvaccinated PLWH showed 7-fold lower neutralization and a higher frequency of nonresponders, with the highest frequency of nonresponders in people with HIV viremia. Vaccinated-only participants showed low neutralization capacity.

**Conclusions:**

The neutralization response of the Delta variant following Ad26.CoV2.S vaccination in PLWH with well-controlled HIV was not inferior to HIV-negative participants, irrespective of past SARS-CoV-2 infection. In SARS-CoV-2—infected and nonvaccinated participants, HIV infection reduced the neutralization response to SARS-CoV-2, with the strongest reduction in HIV viremic individuals.

## Teaser

Ad26.CoV2.S vaccination of people living with well controlled HIV leads to a non-inferior SARS-CoV-2 Delta variant neutralization. In contrast, previously SAR-CoV-2–infected people living with HIV have lower Delta neutralization capacity, which decreases with HIV viremia relative to HIV-negative individuals.

South Africa has a high burden of human immunodeficiency virus (HIV) infection [1] and recent studies observed coronavirus disease 2019 (COVID-19) disease severity [2, 3] and mortality risk [3, 4] are increased among people living with HIV (PLWH). HIV interferes with protective vaccination against multiple pathogens, usually through the decreased effectiveness of the antibody response [5–9]. HIV infection reduces the number of CD4 T cells [10], the primary HIV target cells in different anatomical compartments [11]. Reduced CD4 T-cell numbers correlate with reduced concentrations of antibodies to severe acute respiratory syndrome coronavirus 2 (SARS-CoV-2) [12].

The effects of HIV status on vaccine efficacy are still being determined. While the number of PLWH participants was very small, there was no efficacy of the Novavax NVX-CoV2373 vaccine in PLWH [13]. SARS-CoV-2 vaccine efficacy may also be reduced due to having to cross-neutralize a SARS-CoV-2 variant. For example, infection with the Beta variant [14–16] was associated with a dramatic decrease in the ability the AstraZeneca ChAdOx vaccine to elicit an effective neutralization response [17]. The effect of HIV status on the protection mediated by the adenovirus vectored Ad26.CoV2.S vaccine is yet unknown.

SARS-CoV-2 neutralization by antibodies correlates with SARS-CoV-2 vaccine efficacy [18] and may be a predictor of vaccine efficacy where efficacy data are not yet available. Two studies examining neutralization elicited by the ChAdOx1 nCoV-19 chimpanzee adenovirus vectored vaccine in PLWH with well-controlled HIV observed comparable anti-SARS-CoV-2 spike receptor binding domain (RBD) antibody levels [19, 20]. Decreased neutralization of the ancestral spike sequence in PLWH was observed in 1 study, but 95% confidence intervals (CIs) for neutralization overlapped between PLWH and HIV-negative participants [20]. Interestingly, when neutralization of the Beta SARS-CoV-2 variant was examined in vaccinated participants with detectable neutralization of ancestral virus, 50% of PLWH retained some neutralization activity against the Beta variant compared with only 15% of HIV-negative participants [20].

Several studies examined the effect of HIV on Pfizer-BNT162b2 mRNA vaccine–elicited SARS-CoV-2 spike binding antibodies and neutralization. Most studies found no significant effect of HIV status when testing participants with well-controlled HIV infection [21–24]. One study found that there was also no significant difference in BNT162b2-elicited SARS-CoV-2 binding antibody concentrations to the Beta, Alpha, and Gamma variants in PLWH [24]. A second study found that PLWH with CD4 counts of less than 300 cells/µL (HIV viral load [VL] was unreported) mounted similar anti-SARS-CoV-2 binding antibody responses relative to HIV-negative participants and PLWH with CD4 counts greater than 300 cells/µL [22]. In contrast, another study testing the effect of low CD4 counts showed that anti-SARS-CoV-2 spike receptor binding domain antibodies elicited by BNT162b2 were dramatically lower in PLWH with CD4 counts less than 250 cells/µL [25].

The effect of HIV status on vaccine immunogenicity was examined for the Beijing Institute of Biological Products BIBP-CorV inactivated virus vaccine by measuring binding antibodies and neutralization in a surrogate neutralization assay [26]. This vaccine is administered in 2 doses. Despite the overall conclusion that the vaccine is immunogenic in PLWH, some differences were found. First, PLWH had significantly lower spike RBD binding antibodies after the first (but not the second) dose of the vaccine. Second, PLWH with a CD4:CD8 ratio of less than 0.6, likely indicating HIV-mediated CD4 depletion, showed lower binding and neutralizing antibody responses relative to PLWH with CD4:CD8 greater than 0.6. Whether the participants with CD4:CD8 less than 0.6 were also viremic was not reported. However, about one-third of participants in the study had a detectable HIV VL (defined as >20 HIV RNA copies/mL).

While vaccine elicited neutralization in PLWH vaccinated with the single-dose Johnson and Johnson Ad26.CoV2.S has not been previously reported, data from HIV-negative participants in the SISONKE trial of the Ad26.CoV2.S vaccine in healthcare workers (HCWs) [27] showed moderate neutralization in vaccinated participants, which was enhanced when vaccination was on the background of previous SARS-CoV-2 infection [28].

Here we investigated whether the Ad26.CoV2.S vaccine elicits a comparable neutralizing response to the Delta variant [14] in PLWH relative to HIV-negative study participants using a live virus neutralization assay. We compared the results to SARS-CoV-2-infected unvaccinated participants. The Delta variant was the dominant variant in South Africa and globally at the time when the neutralization assays were performed [14, 29]. We observed that well-controlled HIV infection did not reduce the Ad26.CoV2.S vaccine-elicited neutralization response. In SARS-CoV-2-infected unvaccinated participants, we observed that HIV infection did interfere with the neutralization response to SARS-CoV-2 and interference was strongest in HIV viremic PLWH.

## METHODS

### Ethical Statement

Blood samples were obtained after informed consent from Ad26.CoV2.S vaccinees and adults with polymerase chain reaction (PCR)-confirmed SARS-CoV-2 infection enrolled in a prospective cohort study approved by the Biomedical Research Ethics Committee at the University of KwaZulu-Natal (reference BREC/00001275/2020).

### Cells and Virus Expansion

Vero E6 cells (ATCC CRL-1586, obtained from Cellonex in South Africa) were propagated in complete Dulbecco's modified Eagle medium (DMEM) with 10% fetal bovine serum (Hylone) with 1% each of 4-(2-hydroxyethyl)-1-piperazineethanesulfonic acid (HEPES), sodium pyruvate, L-glutamine, and nonessential amino acids (Sigma-Aldrich). All work with live virus was performed in Biosafety Level 3 containment using protocols approved by the Africa Health Research Institute Biosafety Committee. We used angiotensin-converting enzyme 2 (ACE2)-expressing H1299-E3 cells for the initial isolation (P1 stock) followed by passaging in Vero E6 cells (P2 and P3 stocks, where P3 stock was used in experiments). Viral supernatant was aliquoted and stored at −80°C. The Delta variant virus was isolated as previously described [14]. Detailed information is found in the Supplementary Methods.

### Microneutralization Using the Focus-Forming Assay

Vero E6 cells were plated in a 96-well plate (Corning) at 30,000 cells per well 1 day pre-infection. Plasma was separated from ethylenediaminetetraacetic acid (EDTA)-anticoagulated blood by centrifugation at 500 x *g* for 10 min and stored at −80°C. Aliquots of plasma samples were heat-inactivated at 56°C for 30 minutes and clarified by centrifugation at 10,000 rcf for 5 minutes. GenScript A02051 anti-spike neutralizing monoclonal antibody was added as a positive control to 1 column of wells. Final plasma dilutions were 1:25, 1:50, 1:100, 1:200, 1:400, 1:800, and 1:1600. Virus stocks were used at approximately 50-100 focus-forming units per microwell and added to diluted plasma. Antibody-virus mixtures were incubated for 1 hour at 37°C, 5% CO_2_. Cells were infected with 100 µL of the virus-antibody mixtures for 1 hour, then 100 µL of overlay (1 x Roswell Park Memorial Institute [RPMI] 1640 [Sigma-Aldrich, R6504] with 1.5% carboxymethylcellulose [Sigma-Aldrich, C4888]) was added without removing the inoculum. Cells were fixed 18 hours post-infection using 4% paraformaldehyde (Sigma-Aldrich) for 20 minutes. Foci is stained with a rabbit anti-spike monoclonal antibody (BS-R2B12; GenScript A02058) at 0.5 µg/mL in a permeabilization buffer containing 0.1% saponin (Sigma-Aldrich), 0.1% bovine serum albumin (BSA; Sigma-Aldrich), and 0.05% Tween-20 (Sigma-Aldrich) in phosphate-buffered saline (PBS). Plates were incubated with primary antibody overnight at 4°C, then washed with wash buffer containing 0.05% Tween-20 in.PBS. Secondary goat anti-rabbit horseradish peroxidase (Abcam ab205718) antibody was added at 1 µg/mL and incubated for 2 hours at room temperature with shaking. TrueBlue peroxidase substrate (SeraCare 5510-0030) was then added at 50 µL per well and incubated for 20 minutes at room temperature. Plates were imaged using the ImmunoSpot Ultra-V S6-02-6140 Analyzer Elispot instrument with BioSpot Professional built -in image analysis (Cellular Technology Ltd).

### Multi-Epitope Protein Microarray

ImmuSAFE COVID-19 Array slides (Sengenics Corporation, Singapore) were used to measure the anti-SARS-CoV-2 immunoglobulin G (IgG) antibodies against SARS-CoV-2 nucleocapsid. The microarray-based assays were performed as previously described [30] with modifications as described in the Supplementary Methods. As a threshold, the mean plus 2 standard deviations from pre-pandemic control signal was used.

### Statistics and Fitting

All statistics and fitting were performed using MATLAB v.2019b. Neutralization data were fit to Tx=1/1+(D/ID_50_). Here, Tx is the number of foci normalized to the number of foci in the absence of plasma on the same plate at dilution D and ID_50_ is the plasma dilution giving 50% neutralization: focus reduction neutralization titer (FRNT_50_)=1/ID_50_. Values of FRNT_50_ <1 are set to 1 (undiluted), the lowest measurable value. We note that the most concentrated plasma dilution was 1:25 and therefore FRNT_50_ <25 were extrapolated.

## RESULTS

We tested SARS-CoV-2 neutralization in Ad26.CoV2.S-vaccinated HIV-negative and PLWH participants enrolled in the SISONKE trial, whose aim was to monitor the effectiveness of the single-dose Ad26.COV2.S vaccine among 500 000 HCWs in South Africa (ClinicalTrials.gov number NCT04838795). The SISONKE trial administered only the Ad26.CoV2.S vaccine and started in February 2021. It was the first widespread vaccination effort in South Africa. No other group in addition to HCWs was enrolled. Out of 99 Ad26.COV2.S-vaccinated participants enrolled in our study, 73 (73%) were HIV-negative and 26 (26%) were PLWH. As expected, HCWs are well linked to care and all but 1 vaccinated PLWH showed an undetectable HIV VL (Table 1). As a comparison group, we also enrolled unvaccinated participants with prior documented SARS-CoV-2 infection. This group (n=62) had 28 (45%) HIV-negative participants and 34 (55%) PLWH. In the unvaccinated PLWH group, 29% had a detectable HIV VL, with a median of 3060 (1224-30 160) HIV RNA copies/mL (Table 1). We also used pre-pandemic stored plasma samples as controls (Supplementary Table 1).

**Table 1.** Study Participant Characteristics

	Infected Unvaccinated			Infected and Vaccinated			Vaccinated Only		
	All	HIV−	HIV+	All	HIV−	HIV+	All	HIV−	HIV+
Number of participants	62	28 (45.2%)	34 (54.8%)	67	49 (73.1%)	18 (26.9%)	32	24 (75.0%)	8 (25.0%)
Age, y	44 (39–57)	57 (46–64)	41 (35–45)	46 (40–52)	46 (40–52)	47 (42–51)	45 (39–52)	48 (42–55)	39 (36–42)
Days post-infection	188 (120–278)	192 (108–279)	187 (122–277)	235 (141–306)	230 (134–303)	304 (187–333)	…	…	…
Days post-vaccination	…	…	…	48 (34–81)	48 (34–80)	51 (34–86)	74 (50–84)	74 (44–85)	74 (61–82)
Male sex	12 (19.4%)	5 (17.9%)	7 (20.6%)	2 (3.0%)	2 (4.1%)	0 (0.0%)	1 (3.1%)	0 (0.0%)	1 (12.5%)
Number HIV viremic	…	…	10 (29.4%)	…	…	1 (5.6%)	…	…	0 (0.0%)
HIV viral load	…	…	3060 (1224–30 160)	…	…	3219	…	…	…
Years of ART	…	…	11 (5–15)	…	…	7 (5–12)	…	…	5 (4–11)
CD4 count cells/μL	792 (513–1027)	991 (807–1179)	581 (328–794)	967 (784–1325)	1033 (877–1424)	852 (730–1184)	1199 (853–1368)	1215 (1101–1413)	735 (458–863)
CD4:CD8 ratio	1.1 (0.7–1.2)	1.6 (1.3–2.1)	0.8 (0.4–1.1)	1.6 (1.1–2.2)	1.7 (1.4–2.3)	1.1 (0.8–1.2)	1.8 (1.2–2.1)	1.9 (1.2–2.3)	1.1 (0.4–1.2)

All values are medians (IQR) or n (%). Number HIV viremic is the number of PLWH with HIV RNA >40 copies/mL of total PLWH. Median HIV viral load is for HIV viremic participants only. Abbreviations: ART, antiretroviral therapy; HIV, human immunodeficiency virus. IQR, interquartile range; PLWH, people living with HIV.

We categorized participants into vaccinated only, previously infected and vaccinated, and SARS-CoV-2-infected unvaccinated. The time post-infection of samples from the infection only group was matched as closely as possible to the median time post-infection in the infected/vaccinated group (range, 6-10 months) (Table 1). Vaccination occurred approximately 2 months before blood samples were taken to test neutralization in vaccinated participants (Table 1). We used a record of a SARS-CoV-2-positive quantitative PCR (qPCR) as an indication of previous SARS-CoV-2 infection for all SARS-CoV-2-infected unvaccinated participants and vaccinated participants where such a record was available. To account for asymptomatic or unreported SARS-CoV-2 infection in vaccinated participants, we tested for the presence from SARS-CoV-2 nucleocapsid protein antibodies [30], which are made against the nucleocapsid protein produced in infection but not delivered by Ad26.CoV2.S vaccination. Therefore, a participant was considered previously infected if either nucleocapsid antibodies were detected (Supplementary Figure 1) or a previous positive qPCR for SARS-CoV-2 existed. Of the vaccinated participants, 68% were found to be previously infected with SARS-CoV-2 (Supplementary Figure 1).

The Delta variant became dominant in the province of KwaZulu-Natal, the location of this study, in July 2021 (14). We used a live virus neutralization assay of the Delta variant since it is currently the most widespread variant in South Africa and globally. We note that none of the participants with a record of previous infection were infected in the Delta infection wave (Supplementary Table 2).

**Figure 1.**
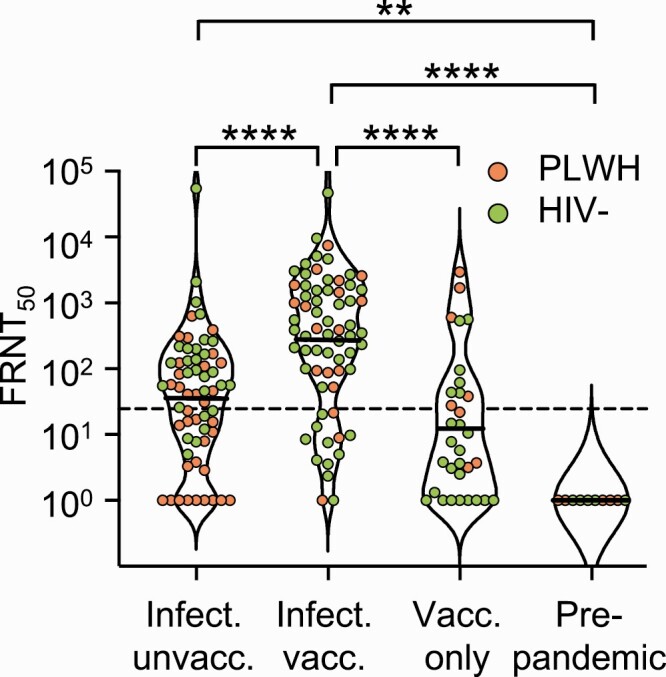
Effect of previous SARS-CoV-2 exposure on neutralization capacity elicited by Ad26.CoV2.S. Violin plots of neutralization capacity of Delta variant as FRNT_50_ in SARS-CoV-2-infected unvaccinated, infected and vaccinated, vaccinated only, and pre-pandemic participants. PLWH are represented by orange points and HIV-negative participants by green points. Horizontal lines represent GMT. Participant numbers per category were n=62 (34 PLWH, 28 HIV) for infected unvaccinated, n=67 (18 PLWH, 49 HIV—) for infected vaccinated, and n=32 (8 PLWH, 24 HIV—) for vaccinated only. *P* values are as follows: ** <.01, **** <.0001 as determined by the Kruskal-Wallis test with Dunn multiple hypothesis correction. The dashed horizontal line denotes most concentrated plasma tested. Abbreviations: FRNT_50_, focus reduction neutralization test (50 is the plasma dilution giving 50% neutralization); GMT, geometric mean titer; HIV, human immunodeficiency virus; Infect., infected; PLWH, people living with HIV; SARS-CoV-2, severe acute respiratory syndrome coronavirus 2; unvacc., unvaccinated; Vacc., vaccinated.

We observed that SARS-CoV-2-infected-only participants had low but detectable SARS-CoV-2 Delta variant neutralization measured in a focus reduction neutralization test (FRNT), where FRNT_50_ is the inverse of the dilution required for 50% neutralization (Figure 1). Neutralization was significantly higher in the group receiving Ad26.CoV2.S vaccination relative to the infected-only group (geometric mean titer [GMT] of 307 [95% CI, 167-562] vs 36 [95% CI, 20.8-63.8], a 9-fold increase; *P* < .0001). Neutralization in the vaccinated/infected group was also 26-fold higher than in the vaccinated-only group (GMT=12 [5.1-28.7], *P* < .0001), although the FRNT_50_ in the latter was below the lowest dilution tested and therefore extrapolated. While neutralization in the infected-only group was higher than in the vaccinated-only group, the difference was not significant.

**Figure 2.**
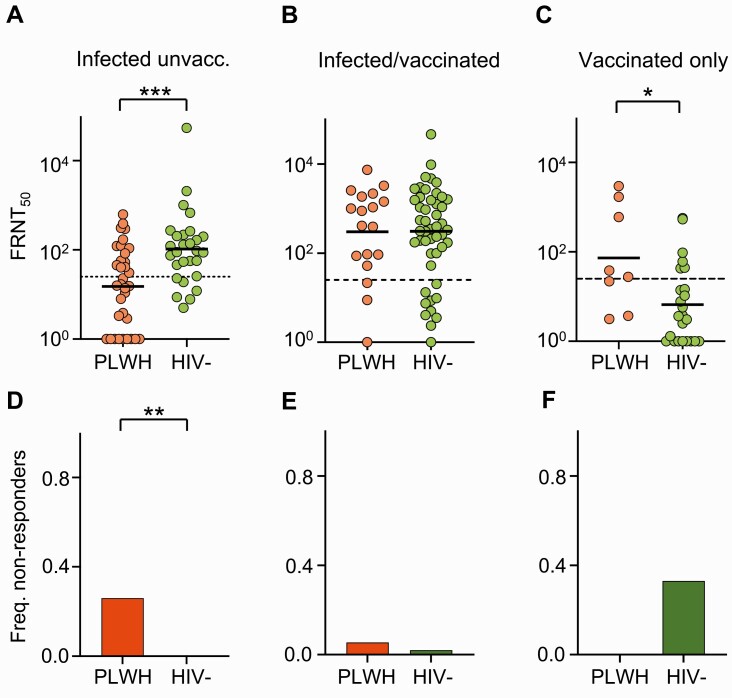
Effect of HIV status on neutralization capacity elicited by Ad26.CoV2.S. (A-C) Neutralization capacity as FRNT_50_ for Delta variant neutralization in SARS-CoV-2-infected unvaccinated (A), infected and vaccinated (B), and vaccinated only (C) participants. Solid horizontal lines represent GMT and dashed horizontal lines represent most concentrated plasma used. *(D-F)* frequency of participants with no detectable Delta variant neutralization (nonresponders) in SARS-CoV-2-infected unvaccinated (D), infected and vaccinated (f), and vaccinated-only (F participants. *P* values are as follows: * <.05, ** <.01, *** <.001, as determined for panels A-C by the Mann-Whitney *U* test and for panels *D-F* by Fisher's exact test. Abbreviations: Freq., frequency; FRNT_50_, focus reduction neutralization test (50 is the plasma dilution giving 50% neutralization); GMT, geometric mean titer; HIV, human immunodeficiency virus; PLWH, people living with HIV; SARS-CoV-2, severe acute respiratory syndrome coronavirus 2; unvacc., unvaccinated.

In the infected-only group, neutralization of the Delta variant was 7-fold lower in PLWH relative to HIV-negative participants (Figure 2A) (GMT=105 [50.4-218] for HIV-negative, 15 [7.3-31.6] for PLWH; *P* = .001). In contrast, there was no significant difference in vaccine-elicited neutralization in PLWH versus HIV-negative participants who received the vaccine following SARS-CoV-2 infection (Figure 2B). In vaccinated-only participants, PLWH seemed to have a stronger vaccine-elicited neutralization of Delta with borderline significance (Figure 2C) (GMT=6 [2.8-15.4] for HIV-negative, 73 [7.9-677] for PLWH; *P* = .02).

We next examined each group for nonresponders, defined as no detectable neutralization of Delta variant neutralization in the live virus neutralization assay (FRNT_50_ =1 in Figure 2A-C). The infected-only PLWH showed a frequency of 26.5% of nonresponders while there were no nonresponders in HIV-negative participants, a significant difference (P=.0029) (Figure 2D). In contrast, the frequency of nonresponders was only 5.6% in PLWH and 2.0% in HIV-negative individuals in the vaccinated/SARS-CoV-2-infected group. The difference between PLWH and HIV-negative participants in the vaccinated, previously infected group was not significant (P=.47) (Figure 2E). In the vaccinated-only group, there were 33.3% nonresponders in the HIV-negative group and none in PLWH, but the difference was nonsignificant (P=.082) (Figure 2F).

**Figure 3.**
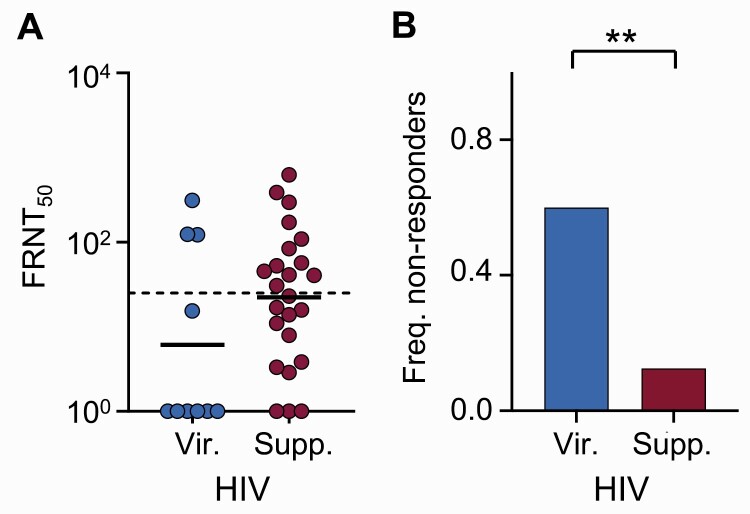
Effect of HIV viremia on neutralization capacity in infected unvaccinated participants. (A) Neutralization capacity as FRNT_50_ for Delta variant neutralization in SARS-CoV-2—infected unvaccinated HIV viremic (n=10) versus infected unvaccinated HIV-suppressed (n=24) participants. The dashed horizontal line represents most concentrated plasma used. (B) Frequency of nonresponders in panel A. *P* values are as follows: *P* = .13 for (A) by the Mann-Whitney *Utest* and *P* = .0088 for (B) by Fisher's exact test. Abbreviations: Freq., frequency; FRNT_50_, focus reduction neutralization test (50 is the plasma dilution giving 50% neutralization); HIV, human immunodeficiency virus; SARS-CoV-2, severe acute respiratory syndrome coronavirus 2; Supp., suppressed; Vir., viremic.

We next determined the effect of HIV suppression in the SARS-CoV-2-infected-only group (the number of HIV viremic participants in the vaccinated groups was too small for analysis). In this group, 29.4% of PLWH participants had detectable HIV viremia (Table 1), compared with 5.6% in the infected/vaccinated group and none in the vaccinated-only group. There was a lower FRNT_50_ in the infected-only viremic versus HIV-suppressed PLWH (GMT, 6 in.HIV viremic vs 22 in suppressed) but this was nonsignificant (Figure 3A) (P=.13). The frequency of nonresponders in the HIV viremic subset was 60.0%, while it was 13.0% in HIV-suppressed PLWH, which was significant (Figure 3B) (P=.0088; odds ratio, 10.5; 95% CI, 1.8-47.0). However, despite HIV suppression by antiretroviral therapy (ART), there was lower neutralization of the Delta variant in SARS-CoV-2-infected-only, HIV-suppressed PLWH relative to HIV-negative participants (Supplementary Figure*2A*), although the difference in the fraction of nonresponders became nonsignificant (Supplementary Figure 25).

**Figure 4.**
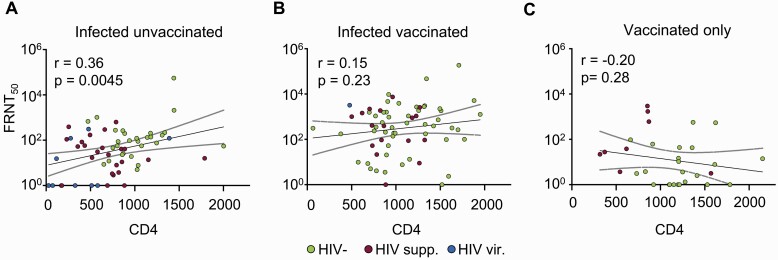
Correlation between CD4 count and neutralization capacity. Pearson correlation of PRNT50 versus CD4 count in infected unvaccinated (A), infected and vaccinated (B), and vaccinated-only (C) participants. Solid lines represent linear regression and upper and lower lines represent 95% confidence intervals. *r* is the Pearson correlation coefficient. Green points are HIV-negative participants, purple points are PLWH with suppressed HIV viremia, and blue points are HIV viremic PLWH. Abbreviations: FRNT_50_, focus reduction neutralization test; HIV, human immunodeficiency virus; PLWH, people living with HIV; supp., suppressed; vir., viremic.

CD4 T-cell count may be an important determinant of the immune response. The CD4 count was lower in the infected-only group (reflecting a higher fraction of PLWH) and was lower in PLWH relative to HIV-negative participants in all groups (Supplementary Figure 3). In the infected-only group, there was a significant correlation between higher CD4 count and higher neutralization (r=0.36, *P* = .0045) (Figure 4A). This correlation was closely associated with HIV status, with the lower CD4 counts being in PLWH. There were no significant correlations between CD4 T-cell count and neutralization in the infected vaccinated or vaccinated-only groups (Figure 4B and 4C).

## DISCUSSION

Our results are consistent with a noncompromised neutralization response to Ad26.CoV2.S vaccination in PLWH. We note that the vaccinated HCW PLWH tested in our study showed well-controlled HIV infection and relatively high CD4 counts. Ad26.CoV2.S uses the ancestral spike sequence. Moreover, all participants with documented previous infection were infected before the emergence of Delta. Therefore, the neutralization capacity we tested was cross-neutralization of Delta by an antibody response elicited to either ancestral spike (vaccine) or ancestral or Beta variant strains (previous infection).

SARS-CoV-2 antibody levels decay post-infection and vaccination after about the first month, with a half-life of approximately 2 months [18]. The interval between infection and sampling was shorter in the infected-only (median, 6.3 months) versus the vaccinated, previously infected (7.8 months) group. It would therefore be expected that infection-elicited neutralization would be higher in the infected-only group if vaccination had no effect. Instead, vaccinated and previously infected participants had 9-fold higher Delta variant neutralization compared with the infected-only group, indicating that vaccination boosted the neutralization response and more than compensated for the longer time post-infection. In the comparison between the vaccinated and vaccinated previously infected group, the vaccinated-only group was sampled later post-vaccination (median, 2.5 vs 1.6 months for vaccinated and infected). However, given a 2-month half-life, the difference in timing does not account for the 26-fold decrease in neutralization in the vaccinated-only group. It is better explained by vaccine boosting of neutralizing immunity acquired through SARS-CoV-2 infection.

The higher neutralization in vaccinated-only PLWH relative to HIV-negative participants was surprising. However, the number of participants in the comparison was small, there was a wide dispersion in FRNT_50_ values, and the vaccinated-only PLWH were younger, perhaps accounting for the better response [31]. Therefore, caution should be used in interpreting these data. A ChAdOx vaccine study previously reported a higher fraction of PLWH participants with well-controlled HIV who detectably cross-neutralized the Beta variant relative to HIV-negative participants, but this, too, was based on low participant numbers [20]. Consistent with results in HIV-negative participants [28], previous SARS-CoV-2 infection enhanced the Ad26.CoV2.S neutralization response.

The effect of HIV status in both the vaccinated-only and vaccinated infected groups contrasts with the infected unvaccinated group, which showed a deleterious effect of HIV infection on neutralization of the Delta variant and an increased number of nonresponders, especially among PLWH with detectable HIV viremia, where the fraction of nonresponders was approximately 5-fold higher than in HIV-suppressed PLWH. However, even in HIV-suppressed PLWH, the neutralization response to Delta was lower. SARS-CoV-2-infected, unvaccinated participants were also the only group where a moderate but significant correlation between CD4 T-cell count and Delta neutralization was detected. We could not examine the effects of HIV viremia on the Ad26.CoV2.S neutralization response in our current study because the SISONKE trial, the first large-scale vaccination effort in South Africa, vaccinated only HCWs, who have good linkage to care and therefore well-suppressed HIV. Future studies will determine the effect of HIV viremia and compare Ad26.CoV2.S with BNT162b2 as the broader population is being vaccinated in South Africa with Ad26.CoV2.S or BNT162b2.

Limitations of this study are that we did not examine the T-cell response or the effect of HIV viremia and low CD4 count on vaccine-mediated neutralization. Also, the number of vaccinated participants without previous SARS-CoV-2 infection, especially in the PLWH group, was small. Both antibody and T-cell responses are critical for effective control and clearance of SARS-CoV-2. Milder COVID-19 disease outcome correlates with a robust T-cell response [32, 33]. If HIV infection dysregulates the T-cell response, it may cause the reported increased COVID-19 disease severity in PLWH [2].

Overall, the results indicate that vaccination with Ad26. CoV2.S has a benefit in terms of conferring SARS-CoV-2 neutralization capacity in PLWH from South Africa with well-suppressed HIV infection.

## Supplementary Material

ciab1008_suppl_Supplementary_Material

## Supplementary Data

Supplementary materials are available at *Clinical Infectious Diseases* online. Consisting of data provided by the authors to benefit the reader, the posted materials are not copyedited and are the sole responsibility of the authors, so questions or comments should be addressed to the corresponding author.

## Notes

***Financial support.*** This work was supported by a South African Medical Research Council Award (6084COAP2020) and the Bill and Melinda Gates award INV-018944 to A. S. D. A. was funded through the South African Medical Research Council (SAMRC) Self-Initiated Grant and the National Research Foundation (NRF) of South Africa Thuthuka (grant number TTK160517165310), the NRF Research Career Advancement Fellowship (grant number RCA13101656388), and a European and Developing Countries Clinical Trials Partnership (EDCTP) senior fellowship (grant number TMA2017SF-1960). J. M. B. received a SARChI grant from the NRF.

***Potential conflicts of interest.*** The authors: No reported conflicts of interest. All authors have submitted the ICMJE Form for Disclosure of Potential Conflicts of Interest. Conflicts that the editors consider relevant to the content of the manuscript have been disclosed.

**COVID-19 Mechanisms and Multi-omics at the Intersection of TB and HIV in KwaZulu-Natal (COMMIT-KZN) Team Members**. Adrie Steyn, University of Alabama at Birmingham, Africa Health Research Institute; Alasdair Leslie, Africa Health Research Institute and Division of Infection and Immunity, University College London; Dirhona Ramjit, Africa Health Research Institute; Emily Wong, Africa Health Research Institute and Division of Infectious Diseases, University of Alabama at Birmingham; Guy Harling, Africa Health Research Institute and the Institute for Global Health, University College London, London, UK; Henrik Kloverpris, Africa Health Research Institute, Division of Infection and Immunity, University College London and Department of Immunology and Microbiology, University of Copenhagen; Jackson Marakalala, Africa Health Research Institute; Janet Seeley, Africa Health Research Institute; Jennifer Giandhari, Kwazulu-Natal Research Innovation and Sequencing Platform; Kaylesh Dullabh, Department of Cardiothoracic Surgery, University of KwaZulu-Natal, Durban, South Africa; Kennedy Nyamande, Department of Pulmonology and Critical Care, University of KwaZulu-Natal, Durban, South Africa; Kobus Herbst, Africa Health Research Institute and the South African Population Research Infrastructure Network, Durban, South Africa; Kogie Naidoo, Centre for Aids Programme of Research in South Africa, University of KwaZulu-Natal; Matilda Mazibuko, Africa Health Research Institute; Moherndran Archary, Department of Paediatrics and Child Health, University of KwaZulu-Natal, Durban, South Africa; Mosa Moshabela, College of Health Sciences, University of KwaZulu-Natal, Durban, South Africa; Nesri Padayatchi, Centre for Aids Programme of Research in South Africa, University of KwaZulu-Natal; Nigel Klein, Africa Health Research Institute and the Institute of Child Health, University College London, London, UK; Nikiwe Mbatha, Africa Health Research Institute.

Nokuthula Ngcobo, Africa Health Research Institute; Nokwanda Gumede, Africa Health Research Institute; Nokwanda Ngcobo, Africa Health Research Institute; Philip Goulder, Africa Health Research Institute and Department of Paediatrics, Oxford, UK; Prakash Jeena, Department of Paediatrics and Child Health, University of KwaZulu-Natal, Durban, South Africa; Rajhmun Madansein, Department of Cardiothoracic Surgery, University of KwaZulu-Natal, Durban, South Africa; Ravindra K. Gupta, Africa Health Research Institute and Cambridge Institute of Therapeutic Immunology & Infectious Disease, Cambridge, UK; Rohen Harrichandparsad, Department of Neurosurgery, University of KwaZulu-Natal, Durban, South Africa; Samita Singh, Africa Health Research Institute; Thandeka Khoza, Africa Health Research Institute; Theresa Smit, Africa Health Research Institute; Thumbi Ndung’u, Africa Health Research Institute, Division of Infection and Immunity University College London, HIV Pathogenesis Programme, The Doris Duke Medical Research Institute and Max Planck Institute for Infection Biology, Berlin; Vinod Patel, Department of Neurology, University of KwaZulu-Natal, Durban, South Africa; Zaza Ndhlovu, Africa Health Research Institute.
