# Supplementary material for: Immunogenicity of Severe Acute Respiratory Syndrome Coronavirus 2 (SARS-CoV-2) Infection and Ad26.CoV2.S Vaccination in People Living With Human Immunodeficiency Virus (HIV)
Source: Clin Infect Dis. 2021 Dec 10;75(1):e857–64. doi: 10.1093/cid/ciab1008 (PMC8689810; doi:10.1093/cid/ciab1008)
Supplement: ciab1008_suppl_Supplementary_Material [file ciab1008_suppl_supplementary_material.pdf]

Table S1: Characteristics of pre-pandemic participants

	All (n=10)	HIV- (n= 5, 50%)	HIV+ (n=5, 50%)
**Age years**	37 (24 - 43)	43 (34 - 44)	24 (24 - 40)
**Male sex**	2 (20.0%)	2 (40.0%)	0 (0.0%)
**HIV viremic**	-	-	3 (60.0%)
**CD4 cells/μL**	666 (398 - 1014)	1046 (853 – 1246.5)	398 (363 - 491)

All values are median (IQR). Fraction HIV viremic is number PLWH with HIV RNA >40 copies/mL of total PLWH.

Table S2: Timing of infection for previously infected participants

	Infection wave 1 (ancestral)	Infection wave 2 (Beta)	Infection wave 3 (Delta)
**Infected**	43	19	0
**Infected/Vaccinated**	25	17	0

Based on date of symptom onset or diagnostic swab for those who did not experience symptoms. Infection wave 1 in South Africa which consisted of ancestral strains of SARS-CoV-2 with the D614G substitution was defined as infections up to 01 November 2020. Infection wave 2 was with the Beta variant and defined as infections between 01 November 2020 to 01 June 2021. Infection wave 3 was with the Delta variant and includes infections from 01 June 2021.

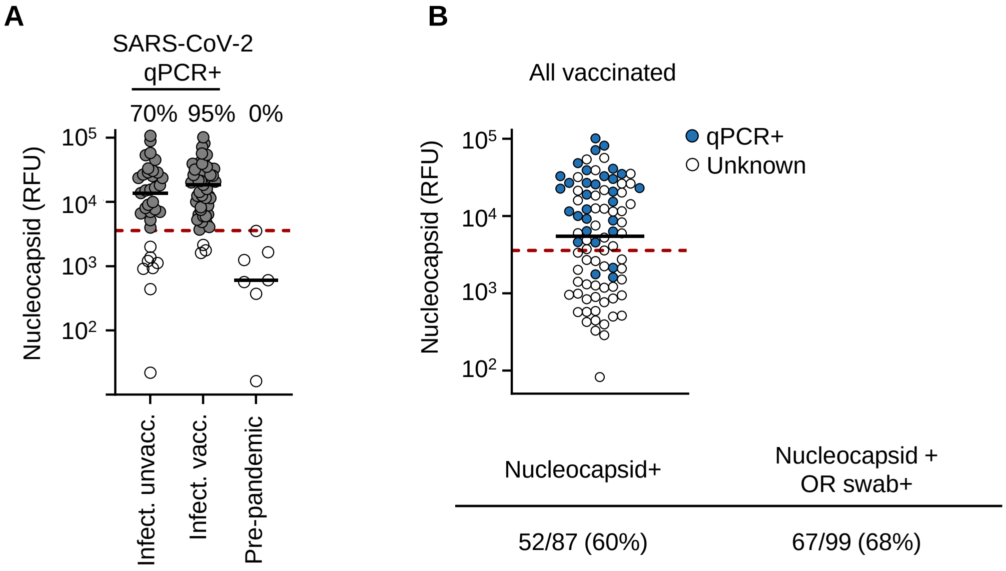

Fig S 1: Determination of previous exposure to SARS-CoV-2 by presence of antibodies to SARS-CoV-2 nucleocapsid. (A) Validation of approach. Blood from 90 infected unvaccinated or infected vaccinated participants who were confirmed SARS-CoV-2 infected by qPCR of a nasopharyngeal/oropharyngeal swab and 6 pre-pandemic controls were tested for antibodies to nucleocapsid. 27 out of 35 (70%) of infected only and 52 out of 55 (95%) of infected and Ad26.CoV2.S vaccinated swab confirmed participants were nucleocapsid antibody positive. All pre-pandemic controls were negative (0/6). (B) Fraction of vaccinated HCW participants with detectable nucleocapsid. Nucleocapsid antibody detection was performed for 87 out of 99 vaccinated participants, with the remaining 12 participants being confirmed for SARS-CoV-2 exposure by qPCR and not included in the nucleocapsid assay. qPCR+: Confirmed by qPCR (blue points). Unknown: no record of positive SARS-CoV-2 PCR (unfilled points). 52 out of 87 (60%) of vaccinated HCW tested were nucleocapsid positive. When the remaining 12 HCW confirmed for SARS-CoV-2 by qPCR were added, the exposure rate increased to 67 out of 99 (68%). Dashed lines in (A) and (B) denote threshold for nucleocapsid detection.

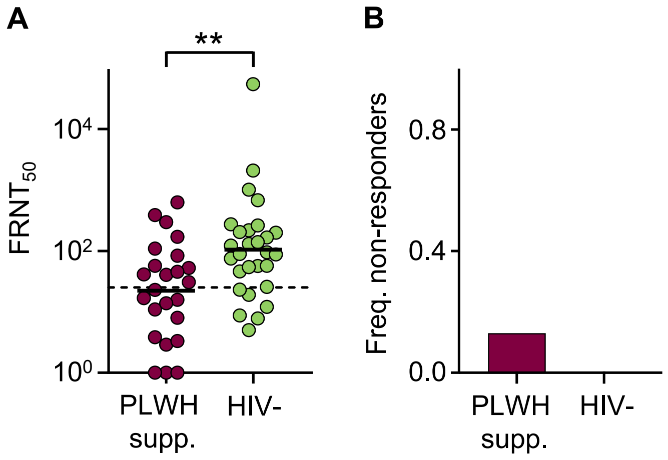

Fig S 2: Differences in Delta neutralization between HIV suppressed and HIV-negative participants. (A) Neutralization capacity as FRNT50 for Delta variant neutralization in SARS-CoV-2 infected unvaccinated participants who are either HIV suppressed (purple points) or HIV-negative (green points). (B) Frequency of non-responders in (A). p-values are p=0.0028, as determined for (A) by the Mann–Whitney U test and p=0.092 for (B) by Fisher's exact test.

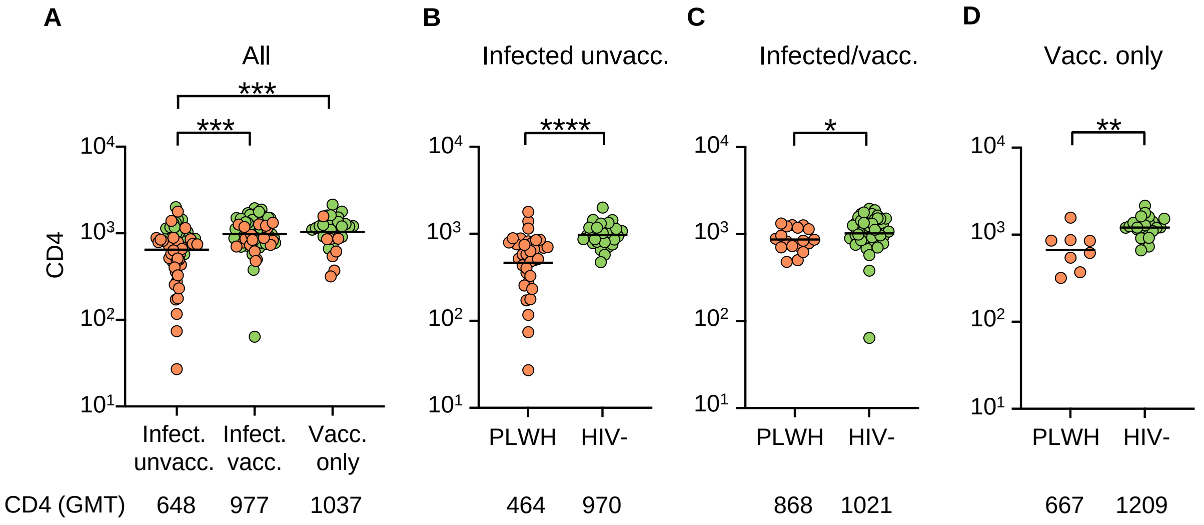

Fig S 3: CD4 T cell counts in PLWH and HIV-negative participants. (A) Comparison of CD4 counts across groups. (B-D) Comparison of CD4 counts between PLWH and HIV-negative participants in the infected unvaccinated (B), infected and vaccinated (C), and vaccinated only (D) groups. The CD4 GMT value is list below each group. Orange points are PLWH, green points are HIV-negative. p-values are * <0.05, ** <0.01, *** < 0.001, **** < 0.0001 as determined by the Kruskal-Wallis test with Dunn multiple hypothesis correction (A) or Mann–Whitney U test (B-D).

Supplementary Methods

Cells

Vero E6 cells (ATCC CRL-1586, obtained from Cellonex in South Africa) were propagated in complete DMEM with 10% fetal bovine serum (Hylone) containing 1% each of HEPES, sodium pyruvate, L-glutamine and nonessential amino acids (Sigma-Aldrich). Vero E6 cells were passaged every 3–4 days. The H1299-E3 cell line for first-passage SARS-CoV-2 expansion was propagated in complete RPMI with 10% fetal bovine serum containing 1% each of HEPES, sodium pyruvate, L-glutamine and nonessential amino acids. H1299 cells were passaged every second day. Cell lines have not been authenticated. The cell lines have been tested for mycoplasma contamination and are mycoplasma negative.

Virus expansion

All work with live virus was performed in Biosafety Level 3 containment using protocols for SARS-CoV-2 approved by the Africa Health Research Institute Biosafety Committee. We used ACE2-expressing H1299-E3 cells for the initial isolation (P1 stock) followed by passaging in Vero E6 cells (P2 and P3 stocks, where P3 stock was used in experiments). ACE2-expressing H1299-E3 cells were seeded at 1.5×10^5 cells per mL and incubated for 18–20 h. After one DPBS wash, the subconfluent cell monolayer was inoculated with 500 μL universal transport medium diluted 1:1 with growth medium filtered through a 0.45-μm filter. Cells were incubated for 1 h. Wells were then filled with 3 mL complete growth medium. After 8 days of infection, cells were trypsinized, centrifuged at 300 rcf for 3 min and resuspended in 4 mL growth medium. Then 1 mL was added to Vero E6 cells that had been seeded at 2×10^5 cells per mL 18–20 h earlier in a T25 flask (approximately 1:8 donor-to-target cell dilution ratio) for cell-to-cell infection. The coculture of ACE2-expressing H1299-E3 and Vero E6 cells was incubated for 1 h and the flask was then filled with 7 mL of complete growth medium and incubated for 6 days. The viral supernatant (P2 stock) was aliquoted and stored at −80 °C and further passaged in Vero E6 cells to obtain the P3 stock used in experiments as follows: a T25 flask (Corning) was seeded with Vero E6 cells at 2×10^5 cells per mL and incubated for 18–20 h. After one DPBS wash, the subconfluent cell monolayer was inoculated with 500 μL universal transport medium diluted 1:1 with growth medium and filtered through a 0.45-μm filter. Cells were incubated for 1 h. The flask was then filled with 7 mL of complete growth medium. After infection for 4 days, supernatants of the infected culture were collected, centrifuged at 300 rcf for 3 min to remove cell debris and filtered using a 0.45-μm filter. Viral supernatant was aliquoted and stored at −80 °C.

Microneutralization using the focus-forming assay

Vero E6 cells were plated in a 96-well plate (Corning) at 30,000 cells per well 1 day before infection. Approximately 5 mL sterile water was added between wells to prevent wells at the edge drying more rapidly, which we have observed to cause edge effects resulting in lower number of foci. Plasma was separated from EDTA-anticoagulated blood by centrifugation at 500 rcf for 10 min and stored at −80 °C. Aliquots of plasma samples were heat-inactivated at 56 °C for 30 min and clarified by centrifugation at 10,000 rcf for 5 min, after which the clear middle layer was used for experiments. Inactivated plasma was stored in single-use aliquots to prevent freeze–thaw cycles. For experiments, plasma was serially diluted and the the GenScript A02051 anti-spike monoclonal antibody was added as a positive control to one column of wells. Final plasma dilutions used were 1:25, 1:50, 1:100, 1:200, 1:400, 1:800, 1:1600 for all plasma samples tested. Virus stocks were used at approximately 50-100 focus-forming units per microwell and added to diluted plasma; antibody–virus mixtures were incubated for 1 h at 37 °C, 5% CO_2. Cells were infected with 100 µL of the virus–antibody mixtures for 1 h, to allow adsorption of virus. Subsequently, 100 µL of a 1X RPMI 1640 (Sigma-Aldrich, R6504), 1.5% carboxymethylcellulose (Sigma-Aldrich, C4888) overlay was added to the wells without removing the inoculum. Cells were fixed at 18 h after infection using 4% paraformaldehyde (Sigma-Aldrich) for 20 min. For staining of foci, a rabbit anti-spike monoclonal antibody (BS-R2B12, GenScript A02058) was used at 0.5 µg/mL as the primary detection antibody. Antibody was resuspended in a permeabilization buffer containing 0.1% saponin (Sigma-Aldrich), 0.1% BSA (Sigma-Aldrich) and 0.05% Tween-20 (Sigma-Aldrich) in PBS. Plates were incubated with primary antibody overnight at 4 °C, then washed with wash buffer containing 0.05% Tween-20 in PBS. Secondary goat anti-rabbit horseradish peroxidase (Abcam ab205718) antibody was added at 1 µg/mL and incubated for 2 h at room temperature with shaking. The TrueBlue peroxidase substrate (SeraCare 5510-0030) was then added at 50 µL per well and incubated for 20 min at room temperature. Plates were then dried for 2 h and imaged using a Metamorph-controlled Nikon TiE motorized microscope with a 2X objective or ELISPOT instrument with built-in image analysis (C.T.L). For microscopy images, automated image analysis was performed using a custom script in MATLAB v.2019b (Mathworks), in which focus detection was automated and did not involve user curation.

Multi-epitope protein microarray

ImmuSAFE COVID-19 Array slides (Sengenics Corporation, Singapore) were used to measure the anti-SARS CoV-2 IgG antibodies against N protein. The microarray-based assays were performed as previously described (Smith et al 2021) with the following modifications. After blocking, the 24-plex microarrays were immediately incubated with the

samples. Heat-inactivated clarified plasma was diluted 1:50 directly in assay buffer (PBST, 0.1% BSA, 0.1% milk powder) and further processed as described (Smith et al 2021). Wells were washed individually three times with 150µl PBST (PBS, 0.2% Tween-20, pH 7.4). Gaskets were then removed, and arrays washed 2 times with 3ml PBST and twice with 3ml PBS. Arrays were then incubated simultaneously with two detection antibodies: AF647 anti-human IgG and AF555 anti-human IgA (ThermoFisher; 1.25µg/ml each in 3ml assay buffer) for 30 min at RT with gentle agitation and dried by centrifugation at 1200× *g* for 2 min. Arrays were then scanned using the InnoScan 710 (Agilent, Santa Clara, CA, USA) fluorescence microarray scanner to generate 16-bit TIFF files. A GAL (GenePix Array List) file containing the identities of each spot was used to extract data from the TIFF images. Data quantification and extraction was automated using Mapix Software (v8.5.0; Innopsys, after which data was analyzed in R Studio. Briefly, for each spot, the neighbourhood background intensity was subtracted from the foreground intensity. As a threshold, the mean plus 2 standard deviations of the background was applied, and negative values zeroed before the mean of triplicate replicate spots was calculated. Any antigens with a co-efficient of variance (CV) > 20% for the technical replicates were flagged, the outlier spots removed and CVs recalculated based on the two remaining spots per antigen. Reciprocal titres were calculated as previously described (Smith et al, 2021). The cumulative tire of the N protein N-terminal domain, C-terminal domain, epitope 1, epitope 2 and epitope 3 was obtained. The thresholds for S and N proteins were determined using the pre-pandemic controls; for the S proteins the mean plus 2x standard deviation of the pre-pandemics were used to set the threshold, whereas for the N proteins the Optimal Cutpoints R package was used, with an emphasis of maximising the specificity (Lopez-Raton et al, 2013).
